# Family Processes and Suicidal Ideation among Chinese Adolescents in Hong Kong

**DOI:** 10.1100/tsw.2011.1

**Published:** 2011-01-05

**Authors:** Sylvia Y. C. L. Kwok, Daniel T. L. Shek

**Affiliations:** ^1^ Department of Applied Social Studies, The City University of Hong Kong, Hong Kong; ^2^ Department of Applied Social Sciences, The Hong Kong Polytechnic University, Hong Kong; ^3^ Public Policy Research Institute, The Hong Kong Polytechnic University, Hong Kong; ^4^ Kiang Wu Nursing College of Macau, China

**Keywords:** Chinese adolescents, family functioning, family processes, parent-adolescent communication, suicidal ideation

## Abstract

Based on the responses of 5,557 Chinese secondary students in Hong Kong, the relationships between perceived family functioning (systemic correlate), parent-adolescent communication (dyadic correlate), and suicidal ideation were examined in this study. Results showed that suicidal ideation was negatively related to global family functioning and parent-adolescent communication. Regression analyses indicated that the dyadic and systemic factors had similar importance in predicting suicidal ideation. Theoretical and practical implications of the findings are discussed.

## INTRODUCTION

### Chinese Conceptions of Suicide

A review of Confucianism, Buddhism, and Taoism as major Chinese religions and philosophies shows that they are opposed to suicidal behaviors[1]. In Confucian thinking, it is maintained that "body, hair and skin come from parents, therefore one dares not to damage" (shen ti fa fu, shou zhu fu mu, bu gan hui shang). In Buddhism, the importance of "nonkilling" (bu xia sheng) of living things (including oneself) is emphasized. Actually, "killing" is one of the acts that Buddhists should avoid as far as possible, while Taoists emphasize the heaven's virtue in preserving life (shun tian er xing).

### Suicide Statistics and Related Phenomena in Hong Kong and the U.S.

Despite these cultural influences, an examination of the suicide statistics indicates that suicide rates in Chinese people are not particularly low. For example, Shek et al.[2] showed that suicide rates in rural China were high and suicide rates among young people in Hong Kong were gradually rising in the 1990s. Adolescent suicidal ideation is also an emerging social problem in Hong Kong in recent years. In two studies of adolescent youths aged 12–18 with 2,427 and 1,309 subjects, 17.8 and 12.8% reported having suicidal ideation, respectively[3,4]. Even more disturbing, another study examining the predictors of suicidal ideation and depression in Hong Kong adolescents reported that 47% of 327 Chinese female students aged 13–18 (Study 1) and 52.6% of 371 Chinese adolescents aged 14–20 (Study 2) had experienced suicidal ideation[5]. The rate of adolescent suicidal ideation in the U.S. is similar. For example, based on the National Youth Risk Behavior Survey conducted in 2005 in the U.S., it was found that 16.9% of 13,953 grades 9–12 students from public and private schools reported suicidal ideation[6]. However, it is noteworthy that there are differences in the suicidal ideation scales used. As suicidal ideation is highly correlated with suicide attempts[7,8], more in-depth study of the correlates of suicidal ideation is essential.

There are studies in Hong Kong and the U.S. showing that family processes are related to adolescent suicidal ideation. In Hong Kong, different studies revealed that an uncaring family environment, negative family climate, low levels of family cohesion and support, high level of parent-adolescent conflict, parental rejection, and too much control were associated with adolescent suicidal thoughts[5,9,10]. Similarly, previous studies in the U.S. found that adolescent suicidal ideation was positively related to family dysfunction, family discord, poor family environment, family rigidity, family conflicts, and poor adaptability, but negatively related to perceived parental support and family support[11,12,13].

Although similar research findings in the U.S. and Hong Kong are reported, there are two significant areas of difference. First, research findings show that Hong Kong adolescents are more dependent on their families for emotional and instrumental support than adolescents in the U.S.[14]. As such, it is interesting to ask whether the influence of family processes on adolescent suicidal ideation would be different within Hong Kong. Second, as the subjects being studied are Chinese adolescents living in Hong Kong, they are under the strong influence of Chinese culture. Hong Kong is regarded as a Chinese society and the families in Hong Kong are usually referred to as Chinese families. Since Chinese families have some characteristics that are different from those of Western families, e.g., emphasis on harmony and nonexpression of emotions[15], it would be interesting to study how family processes are related to adolescent suicidal ideation in Hong Kong families.

## Gaps in the Existing Studies Related to Family Processes and Suicidal Ideation

First, the existing findings on the relationship between family functioning and adolescent suicidal ideation are equivocal. For example, a study in the U.S. by Mitchell and Rosenthal[16] found no significant group difference in terms of the mean family rigidity scores in the families of suicidal and nonsuicidal psychiatric inpatient adolescents. In addition, no association between perceived parent-adolescent relationship and adolescent suicidal behavior was found in another U.S. study[17].

Second, although there are differences in the cultural norms for “healthy” family functioning between Western and Chinese societies, there are very few studies utilizing indigenous Chinese measures of family functioning. According to a study on the attributes of happy families in Hong Kong[15,18], Chinese parents and their children regarded the absence of conflict, interpersonal harmony, mutuality, connectedness, and positive parent-adolescent relationships as important attributes of a happy family. Although contemporary Western societies emphasize mutual respect among family members, Chinese families are more hierarchical in structure[19,20]. For example, Chinese wives are taught to be submissive to their husbands and children are socialized to obey their fathers, in accordance with the *wu lun* in traditional Chinese culture[21]. In view of the difference between Western and Chinese cultures, it would be theoretically important to ask whether the hypothesized negative relationship between family functioning and adolescent suicidal ideation can be found in the Chinese culture.

Third, few studies have been conducted to examine parent-adolescent communication and adolescent suicidal ideation both in the U.S. and Hong Kong[22,23]. Even given the limited number of studies, there are inconsistent findings, particularly with respect to gender and parental differences in the linkage between parent-adolescent communication and suicidal ideation. For example, while Adams et al.[24] showed that perceived problems in communication with the mother, but not with the father, differentiated suicidal adolescents from nonsuicidal adolescents, King et al.[25] showed that suicidal adolescents reported less active and communicative relationships with the fathers, but not with the mothers, in the U.S. Basically, one should ask whether the linkages between these two domains are different in father-son, father-daughter, mother-son, and mother-daughter dyads. Studies in the U.S. and Hong Kong showed a stronger impact of parents on girls than on boys[26,27], while mother-adolescent relationships had a stronger impact on adolescent developmental outcomes than did father-adolescent relationships[28,29]. Hence, whether mother-adolescent communication exerts a stronger influence on adolescent suicidal ideation than does father-adolescent communication should be examined.

Fourth, most of the measuring instruments on parent-adolescent communication for the Hong Kong studies were adapted from the West, which may not be able to capture the essence of the Chinese culture. In the traditional Chinese culture, open parent-adolescent communication was basically not encouraged because children were taught to inhibit their expression of feelings and exhibit a general pattern of respect, obedience, and piety[30]. Within such a cultural context, this repression of feelings and opinions should also be included in analyzing the parent-adolescent communication process. Hence, one should develop indigenous measures of parent-adolescent communication in order to examine the related issues with reference to the uniqueness of the Chinese culture.

Fifth, it is not clear which family process, if any, is comparatively more important than others. Within the family system, three basic processes are present, including individual process (e.g., development of resilience), dyadic process (e.g., parent-adolescent communication), and systemic process (e.g., family functioning). Previous findings on their relative importance are equivocal. A study found that after controlling for other causal factors (e.g., family adaptability and cohesion), poorer parent-adolescent communication remained strongly associated with adolescent self-harm[31]. On the contrary, another study showed that family strengths had an indirect effect on adolescent depression through self-esteem, while parent-adolescent communication did not have a direct effect or an indirect effect on adolescent depression[32]. Although some studies stressed both parent-adolescent communication and family functioning as important predictors of adolescent suicidal ideation[33], it is theoretically important to ask whether a systemic or dyadic mechanism is more important for predicting suicidal ideation. For example, as family communication involves interaction among different family members, including grandparents and siblings, while parent-adolescent communication includes only the parent-adolescent dyad, their effects on adolescent suicidal ideation may be different.

## Conceptual Framework of the Study

The family systems theory[34] is adopted in this study. As the family is a system composed of interconnected and interdependent individuals, a person cannot be understood in isolation from the family system. An individual’s behavior is caused by interaction with other family members and dynamics within the family system as a whole. Hence, to understand adolescent suicidal ideation, it is important to examine the family processes that include the adolescents’ interaction with the parents (i.e., dyadic process, parent-adolescent communication) and the impact of the family system (i.e., systemic process, family functioning) on their thinking and behavior. The systems perspective has been used to look at the relationship among individual, dyadic, and systemic processes in the family. For example, Shek[29] adopted the systems perspective to understand how individual and dyadic processes contribute to systemic family functioning. Based on the family systems perspective, a conceptual model was formulated and several research questions were addressed in this study (Fig. 1).

## Research Questions and Hypotheses

The first research question is: What is the relationship between parent-adolescent communication and Chinese adolescent suicidal ideation in Hong Kong? Based on the family systems theory that child development is affected by interaction with their parents[34] and previous research findings[35], it was

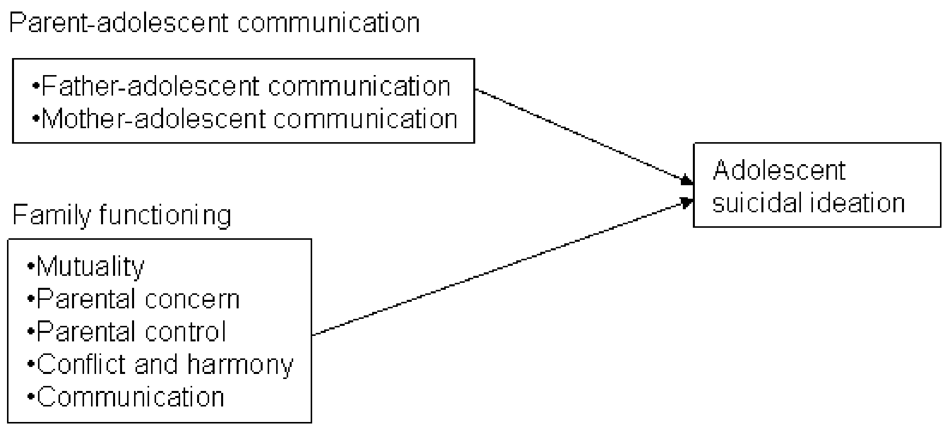

**FIGURE 1.** Conceptual framework on the relationship between parent-adolescent communication and family functioning and adolescent suicidal ideation.

hypothesized that parent-adolescent communication would have a negative relationship with adolescent suicidal ideation (Hypothesis 1). With reference to previous studies[26,28], it was hypothesized that mother-adolescent communication would have a more significant relationship than father-adolescent communication with suicidal ideation, especially for female adolescents (Hypothesis 1a). Furthermore, as research findings showed that fathers exerted stronger influence on daughters than on sons, it would be expected that father-adolescent communication would have a stronger association with suicidal ideation in daughters relative to sons (Hypothesis 1b).

The second question is: What is the relationship between perceived family functioning and adolescent suicidal ideation? Based on the prediction of family systems theory that individuals are affected by the family system as a whole[34] and previous research findings[13,36], it was hypothesized that perceived family functioning would have a negative relationship with adolescent suicidal ideation (Hypothesis 2).

The third research question is: Are different dimensions of family functioning (i.e., mutuality, communication, conflict and harmony, parental control, parental concern) differentially related to adolescent suicidal ideation? Based on Western theories of family functioning (e.g., Beaver’s and Olson’s models) derived from family systems theory, communication is a central family process. As such, it was expected that relative to other aspects of family functioning, communication would have the greatest effect on adolescent suicidal ideation (Hypothesis 3a). On the other hand, as Chinese families emphasize the importance of maintaining harmony, avoiding conflict, and promoting family cohesion and mutual support, it was alternatively postulated that “conflict and harmony” would be the most important predictor, followed by mutuality, communication, parental concern, then parental control (Hypothesis 3b).

The fourth research question is: What is the relative importance of parent-adolescent communication and family functioning in predicting adolescent suicidal ideation? As previous studies yielded controversial results, it is theoretically and practically interesting to explore the relative importance of dyadic communication and systemic family functioning in predicting adolescent suicidal ideation.

## METHODS

### Participants and Procedures

A cross-sectional survey was conducted and convenience sampling was used. School social workers helped to contact the principals of the secondary schools in which they were stationed. Among the principals of 68 schools (out of a total of 426 secondary schools in Hong Kong) contacted, those from 42 schools (including self-financed, subvented, and government schools) from Hong Kong Island, Kowloon, and the New Territories agreed to join the study. The response rate was 62%, which is comparable with other similar studies on suicidal ideation and behavior[37,38]. By the end of the survey period, a total of 5,557 valid questionnaires had been collected. With reference to the total secondary student population in Hong Kong at the time the study was conducted (328,573 students), the sample size used (2% of the total secondary student population) can be regarded as adequate, and the gender and form ratios in the sample were similar to those based on the population. There were slightly more males (53.1%) than females (46.9%) in the sample, which reflects the distribution among the general population. The participants were evenly distributed among different forms (25.8% in Grade 7, 24.7% in Grade 8, 24.4% in Grade 9, 25.1% in Grade 10). The age of the respondents varied from 11 to 18 years, with an overall mean of 13.87 years (*SD* = 1.47).

Parental consent letters were sent to the parents to explain the purpose of the study and get their consent prior to the survey. During the survey, a school social worker, a teacher, or a research assistant was present to give a short briefing on the general aims and the confidentiality of the study. It was clearly stated that participation in the study was voluntary and they could refuse to complete the questionnaires if so desired. Refusal to join the study would carry no subsequent penalty. Consent forms were signed by those participating students. The research assistants, but not the teachers nor the social workers, were present throughout the whole administration process to answer queries raised by the participants. The administration process took around 40 min to complete. Since the questionnaires were anonymous, individual participants could not be identified. To facilitate follow-up work by the school social workers, individual school reports were sent to the workers so that they could identify those classes that had more students with higher suicidal ideation in order to hold prevention groups and programs.

## Instruments

- **Parent-adolescent communication**. Parent-adolescent communication was assessed using the Father-Adolescent Communication Scale (FACS) and Mother-Adolescent Communication Scale (MACS) developed by Shek et al.[23]. Each instrument contains 25 identical items and assesses the quality of parent-adolescent communication with either the father or mother (e.g., “I can voice out my thinking and feeling to my father/mother in our communication”; “Father/mother knows my feeling even when he/she has not asked me”). As the Chinese are not accustomed to expressing their feelings, there are eight items in the scale to explore this phenomenon descriptively. A higher FACS or MACS score indicates a higher level of quality of parent-adolescent communication. Moreover, Shek et al.[23] reported that the two measures were able to discriminate adolescents with good and bad communication with their parents, thus lending support to the criterion-related validity of the measure. The scale had significant correlation with adolescent psychological well-being (i.e., mastery and life satisfaction). Reliability analyses showed that the FACS and MACS had high reliability in this study ($\alpha$ = 0.93 and 0.94, respectively).
- **Family functioning**. Family functioning was assessed using the Chinese Family Assessment Instrument (C-FAI), which is one of the first indigenous instruments designed to measure family functioning in Chinese families[39]. For each question, respondents were required to choose an answer from a 5-point Likert scale. There are five subscales: Mutuality (e.g., family members accommodate each other), Communication (e.g., family members enjoy getting together), Conflict and Harmony (e.g., there is much friction among family members), Parental Concern (e.g., parents take care of their children), and Parental Control (e.g., parents force children to do things). A higher score in the five subscales indicates a higher level of family functioning. On closer examination of the dimensions of the C-FAI, the Mutuality and Communication factors represent dimensions related to cohesiveness and communication in Western literature. The Conflict and Harmony dimension was a distinct dimension of family functioning in Hong Kong Chinese families, reflecting the importance attributed to the “absence of conflict”, which was regarded by the Chinese parents and children as an attribute of happy families in Hong Kong[28]. The other two dimensions, Parental Concern and Parental Control were related to functioning of parents, which suggested that parents were quite influential in family functioning in the Chinese families. A series of large-sample validation studies were conducted[40,41], which provided strong support for the psychometric properties of the scale. It was found that there was moderate correlation between C-FAI and parent-adolescent conflict measured by the Conflict Behavior Questionnaire[42]. The scale was demonstrated to have high reliability in this study ($\alpha = 0.93$).

- **Suicidal ideation**. Adolescent suicidal ideation was assessed using the Suicidal Ideation Sub-Scale (C-SIS) of the Suicidal Risk Scale for Hong Kong students developed by Tse[43]. The instrument is used as a screening tool to identify high-risk students so that effective intervention can be implemented. In addition, it is used for assessment of suicidal risk during the intervention phase, and for evaluation of the effectiveness of intervention and treatment programs. The C-SIS contains 13 items (e.g., "I really want to put an end to all this so that I don't have to continue to bear the pain"; "Some problems can only be solved by death"). The Chinese tend to use escape and avoidance when facing problems, and might regard death as the only way out when the problem could not be solved[44]. For each item, respondents were required to choose an answer from a 4-point Likert scale ranging from "*strongly agree*" to "*strongly disagree*". A higher score indicates a higher level of suicidal ideation. Previous findings based on a validation study[43] showed that the C-SIS has high internal consistency ($\alpha = 0.92$), split-half consistency ($\alpha = 0.88$), and test-retest reliability ($r = 0.72$). In the study of construct validity by Tse and Bagley[43], C-SIS displayed significant relationships with other related measures such as Suicide Behavior Questionnaire[45] and the CES-D[46]. Moreover, the C-SIS scores were able to discriminate suicidal and nonsuicidal students, with a significant t-value of –23.26 ($p < 0.001$). Reliability analyses showed that the scale had high reliability in this study ($\alpha = 0.93$).
- **Parents' demographics** As previous studies demonstrated that parents' education level and family income were significant predictors of adolescent suicidal ideation[47,48], they were incorporated as covariates in hierarchical regression analyses of the current study.

## RESULTS

The mean total scores for the FACS, MACS, C-FAI, and C-SIS were 60.45 ($SD = 13.49$), 69.27 ($SD = 13.92$), 118.72 ($SD = 24.34$), and 22.54 ($SD = 8.64$), respectively. Although there was no clinical cutoff score for C-SIS, about 9.3% of the sample had a raw score of 35 or above, which was a relatively high score as defined by Tse's study[43]. Hence, a score of 35 or above indicates high levels of suicidal ideation in this study.

Pearson correlation analyses showed that there was significant correlation among father-adolescent communication, mother-adolescent communication, family functioning, and male and female adolescent suicidal ideation (Table 1).

## Research Question 1

Pearson correlation analyses supported Hypothesis 1 that perceived parent-adolescent communication would have a negative relationship with adolescent suicidal ideation. The relationship between mother-adolescent communication and suicidal ideation was higher than father-adolescent communication and suicidal ideation in the male sample ($t = 4.76$, $p < 0.001$) and the female sample ($t = 4.34$, $p < 0.001$) (Table 1), thus providing some support for Hypothesis 1a. Further analysis showed that the relationship between father-adolescent communication and suicidal ideation in the male respondents was weaker than that in the female respondents ($t = 2.99$, $p < 0.01$), which gives support to Hypothesis 1b.

**TABLE 1**
**Correlations among Father-Adolescent Communication, Mother-Adolescent Communication, Family Functioning, and Adolescent Suicidal Ideation Split by Gender**

| | Father-Adolescent Communication | Mother-Adolescent Communication | Family Functioning | Adolescent Suicidal Ideation |
|---|---|---|---|---|
| **Father-Adolescent Communication** | — | 0.37*** | 0.65*** | –0.39*** |
| **Mother-Adolescent Communication** | 0.41*** | — | 0.67*** | –0.43*** |
| **Family Functioning** | 0.66*** | 0.66*** | — | –0.48*** |
| **Adolescent Suicidal Ideation** | –0.33*** | –0.42*** | –0.43*** | — |

***$p$ < 0.001, correlation coefficients for females above diagonal and males below diagonal.

Moreover, hierarchical regression analyses were performed. Demographic variables (including fathers' and mothers' education, monthly family income), parent-adolescent communication (including father-adolescent communication, mother-adolescent communication), and family functioning (including mutuality, communication, conflict and harmony, parental concern, parental control) were entered into hierarchical blocks. It was found that the fathers' education was a significant predictor of adolescent suicidal ideation, although the related effect size was small. It was also shown that father-adolescent communication ($\beta$ = –0.13, $p$ < 0.001) and mother-adolescent communication predicted adolescent suicidal ideation ($\beta$ = –0.23, $p$ < 0.001), explaining 22% of the variance in the total sample when the demographic characteristics were controlled (Table 2). However, some gender and parental differences in the relationship between parent-adolescent communication and suicidal ideation were identified. First, father-adolescent communication exerted a stronger effect on daughters ($\beta$ = –0.16) than sons ($\beta$ = –0.10), $z$ = 3.71, $p$ < 0.001. Second, parental differences in the linkage between the two domains were found. In the male respondents, mother-son communication had a stronger linkage with suicidal ideation than did father-son communication ($t$ = 8.54, $p$ < 0.001). Among the female respondents, mother-daughter communication similarly had a stronger linkage with suicidal ideation than did father-daughter communication ($t$ = 4.69, $p$ < 0.01). These findings provide support for Hypothesis 1b.

## Research Question 2

Pearson correlation analyses with Bonferroni-corrected alpha levels were performed. There was also support for Hypothesis 2 in which perceived family functioning would be negatively related to adolescent suicidal ideation. Specifically, adolescent suicidal ideation was negatively related to mutuality, communication, conflict and harmony, parental concern, and parental control ($r$ = –0.39, –0.36, –0.44, –0.39, –0.39, $p$ < 0.0083) (Table 3).

## Research Question 3

Analyses showed that the level of multicollinearity within different family predictors of adolescent suicidal ideation is acceptable in the current study, with all variation inflation factors (VIFs) below 10[49]. Among the five dimensions of family functioning, hierarchical regression analysis showed that mutuality, conflict and harmony, parental concern, and parental control were significant predictors of adolescent suicidal ideation (Table 2). Contrary to the prediction of Western family theories, communication

**TABLE 2**
**Hierarchical Multiple Regression Analysis for the Prediction of Adolescent Suicidal Ideation in the Total Sample**

| | Step 1 ($\beta$) | Step 2 ($\beta$) | Step 3 ($\beta$) |
|---|---|---|---|
| Fathers' education[a] | –0.06** | –0.03 | –0.03 |
| Mothers' education | 0.002 | 0.03 | 0.02 |
| Family income | –0.03 | –0.002 | 0.01 |
| Father-adolescent communication | | –0.23*** | –0.13*** |
| Mother-adolescent communication | | –0.35*** | –0.23*** |
| Mutuality | | | –0.10** |
| Communication | | | –0.04 |
| Conflict and harmony | | | –0.16*** |
| Parental concern | | | –0.14*** |
| Parental control | | | –0.08*** |
| $F$ | 8.20 | 205.45 | 144.33 |
| $R^2$ | 0.01 | 0.23 | 0.28 |
| Change in $R^2$ | | 0.22 | 0.05 |

*Note:* $\beta$ = Standardized regression coefficient.

[a] Fathers' education (1 = primary education or below, 2 = secondary education, 3 = tertiary education or above).

**$p$ < 0.01, ***$p$ < 0.001.

**TABLE 3**
**Correlation Coefficients on the Relationships between Measures of Family Functioning and Adolescent Suicidal Ideation**

| Suicidal Ideation | Mutuality | Communication | Conflict and Harmony | Parental Concern | Parental Control | Total |
|---|---|---|---|---|---|---|
| Male | –0.36* | –0.33* | –0.44* | –0.40* | –0.38* | –0.43* |
| Female | –0.43* | –0.39* | –0.44* | –0.39* | –0.41* | –0.48* |
| Total | –0.39* | –0.36* | –0.44* | –0.39* | –0.39* | –0.46* |

*$p$ < 0.0083 (Bonferroni–corrected alpha level).

was not a significant predictor (i.e., no support for Hypothesis 3a). Further analyses showed that there were no significant differences among the betas of different dimensions of family functioning and suicidal ideation (Table 4). Hence, mutuality, conflict and harmony, parental concern, and parental control had similar importance in predicting suicidal ideation (i.e., no support for Hypothesis 3b). In addition, it was shown that family functioning predicted an additional 5% of the variance in the total sample.

## Research Question 4

A comparison of the regression coefficients of the dyadic and systemic variables showed that both family functioning ($t$ = 6.06, $p$ < 0.001) and mother-adolescent communication ($t$ = 7.96, $p$ < 0.001) were stronger

**TABLE 4**
**Comparison of the Strength of Relationships (Beta Coefficients) between Different Dimensions of Family Functioning and Suicidal Ideation**

| | Mutuality & SI | Conflict and Harmony & SI | Pconcern & SI | Pcontrol & SI |
|---|---|---|---|---|
| **Mutuality & SI** | — | | | |
| **Conflict and Harmony & SI** | –0.10 vs. –0.16, t = 1.70 | — | | |
| **Pconcern & SI** | –0.10 vs. –0.14, t = 0.92 | –0.16 vs. –0.14, t = 0.41 | — | |
| **Pcontrol & SI** | –0.10 vs. –0.08, t = 0.28 | –0.16 vs. –0.08, t = 1.79 | | — |

*Note:* SI = suicidal ideation; Pconcern = parental concern; Pcontrol = parental control.

**TABLE 5**
**Comparison of the Strength of Association (Beta Coefficients) between Dyadic and Systemic Family Processes and Adolescent Suicidal Ideation**

| | Family Functioning & SI | Father-Adolescent Communication & SI | Mother-Adolescent Communication & SI |
|---|---|---|---|
| **Family Functioning & SI** | — | | |
| **Father-Adolescent Communication & SI** | –0.22 vs. –0.13, t = 6.06*** | — | |
| **Mother-Adolescent Communication & SI** | –0.22 vs. –0.23, t = 1.36 | –0.13 vs. –0.23, t = 7.96*** | — |

****p* < 0.001.

predictors of suicidal ideation than was father-adolescent communication, while there were no significant differences between mother-adolescent communication and family functioning in predicting suicidal ideation (Table 5). Hence, both dyadic (at least for mother-adolescent communication) and systemic factors have similar importance in predicting adolescent suicidal ideation.

## DISCUSSION

In response to the limitations in the related literature, there are several unique features of the present study. First, Chinese adolescents were recruited in this study. Second, a large sample based on students in Grades 7–10 was employed. Third, locally developed and validated measures of parent-adolescent communication, family functioning, and suicidal ideation were used. Fourth, the relationship between father-son, father-daughter, mother-son, and mother-daughter communications and adolescent suicidal ideation was explored, while the relative impact of father-adolescent communication and mother-adolescent communication on adolescent suicidal ideation was examined. Fifth, the relative importance of different dimensions of family functioning on adolescent suicidal ideation was studied. Finally, the relative importance of the dyadic and systemic processes in predicting adolescent suicidal ideation was examined. Practically, it has implications on the different roles played by fathers and mothers in preventing adolescent suicidal ideation, and how adolescents with suicidal ideation should be treated (e.g., family therapy or individual counseling).

Results indicated that the fathers' education impacted adolescent suicidal ideation, which corroborates previous findings[47]. Fathers with better education were found to be more confident in parenting, more respectful of their children, and more warm and involved in child rearing. Since suicidal adolescents perceived their parents to be significantly more critical and less caring, educated parents who were more caring would inevitably have a better relationship with their children, which could have a positive impact on the adolescents' psychological well-being and lower their suicidal ideation.

Both correlation and regression analyses showed that a higher level of parent-adolescent communication was significantly related to a lower level of adolescent suicidal ideation, thus giving support for Hypothesis 1. This observation is in line with research findings showing that there were less frequent and less satisfying parent-adolescent communications among suicidal adolescents[50]. Moreover, this observation is consistent with the prediction of family systems theory[34] that parent-child interaction is a determinant of a child's psychological well-being.

When the male and female respondents were compared, father-adolescent communication appeared to have a stronger impact on girls than boys, thus supporting Hypothesis 1a. This finding is consistent with research findings in the U.S. Herrara et al.[51] reported that the absence of the father in the family contributed strongly to the daughters' suicidal behavior. Both U.S. and Hong Kong studies showed that adolescent girls were more susceptible to the influence of family than were adolescent boys (e.g., [26,27]). Due to the difference in communication styles with the boys, girls may like to express themselves more and share their problems with their fathers, who may stimulate them to think out solutions with different perspectives. Contrarily, boys are more reserved and are less likely to share things and problems with others, especially the same-sex fathers. Therefore, negative parent-adolescent communication would exert a stronger impact for girls than for boys. This finding suggests that the gender of children should be taken into account in order to understand the impact of fathers on child developmental outcomes.

For both male and female respondents, mother-adolescent communication correlated more highly with suicidal ideation than father-adolescent communication, which gives partial support to Hypothesis 1b. This is in line with previous studies in which suicide attempters reported significantly less mutuality and communication with their mothers than their fathers (e.g., [52]). Traditionally, fathers performed the breadwinner role, while mothers played the socialization and caregiving roles in Chinese society. These traditional gender role differences contribute to the mothers spending more time with the children, while fathers spend more time at work. Furthermore, Chinese women are more encouraged to express their thinking and emotions, and are better listeners than men. Hence, adolescents like to communicate with their mothers more than their fathers. These cultural conditions may explain why mother-adolescent communication was regarded as more important for the adolescents than father-adolescent communication[23]. Theoretically speaking, the findings underscore the complexity of the relationship between parent-adolescent communication and adolescent suicidal ideation, and highlight that gender and parental differences should be taken into account. Furthermore, it is possible that parents may have different impacts on adolescents, depending on the nature of outcome indicators under focus.

Results also showed that perceived healthy family functioning is negatively related to adolescent suicidal ideation, supporting Hypothesis 2. This is consistent with the prediction of the family systems theory that family functioning is closely linked to a child's psychological well-being[34]. It also corroborates the previous finding that poorer family functioning was significantly correlated with higher suicidal ideation[53]. Among the five dimensions of family functioning, mutuality is one of the significant predictors of adolescent suicidal ideation. As Chinese families emphasize family cohesion, mutual trust, and support among the family members, adolescents living in families lacking mutuality may feel lonely and frustrated, thus increasing their suicidal ideation.

With reference to Hypothesis 3a, it is interesting to note that communication in the whole family was not a significant predictor for adolescent suicidal ideation. This is not consistent with the prediction of Western family models (e.g., [36,54]) and previous studies[23] that emphasize the role of communication within the family. This puzzle may be due to the methodological characteristic of the study. Some of the items in the instrument asked the respondents whether their family members usually arranged family activities, enjoyed getting together, and talking to one another. As the adolescents at their developmental stage usually enjoyed getting together and having activities with their peers rather than family members, family activities might not be regarded as important. This explains why communication in the family is not an important predictor of adolescent suicidal ideation.

The finding that conflict and harmony significantly predicts adolescent suicidal ideation is in line with previous findings[10,55]. Traditionally, Chinese people emphasized avoidance of interpersonal conflict and cultivation of interpersonal harmony, as revealed in the Chinese cultural sayings, "If a family lives in harmony, everything will prosper. A family will wither if there are many quarrels." Confucianism emphasized social and family harmony[56,57]. Chinese families were generally thought to be highly cohesive, partially due to a strong cultural emphasis on harmony and mutual obligations, with low value placed on overt expressions of affection and disapproval. Obedience and respect for the authority were valued and conflicts in the families were suppressed[58]. Hence, overt conflict in the family, particularly parent-adolescent conflict, would cause disturbance and stress to the adolescents, affecting their psychological well-being (e.g., sense of hope, satisfaction with life), thus increasing their suicidal ideation[27]. However, the findings did not provide support for Hypothesis 3b.

Results of the study showed that parental concern was also a strong predictor of adolescent suicidal ideation. As adolescents experienced a period of storm and turmoil, they still needed a lot of emotional support from their parents. Hence, parental concern was important and positively related to the adolescents' psychological adjustment and well-being[59], which would also affect their suicidal ideation. Furthermore, parental control was another important predictor of adolescent suicidal ideation. It is understandable that adolescents, who strive for freedom and independence, would resist parental control. Previous studies showed that the adolescent suicide attempters perceived their parents to be less affectionate and more controlling with them than their siblings (e.g., [60]). A Hong Kong study also found that parental psychological control was negatively related to adolescent psychological well-being[61]. Hence, parental control would affect the adolescents' psychological well-being, which contributed to suicidal ideation.

When comparing dyadic and systemic impacts with reference to research question 4, it was found that both mechanisms have similar predictive power on adolescent suicidal ideation. Although general family functioning affects adolescents, parent-adolescent communication has similar impacts. In Chinese families, parent-child communication and relationships are emphasized more than sibling or other familial relationships. Positive encouragement, support, and concern from parents are more treasured by the children, although family support, cohesion, and harmony also play an important role in a child's development. Hence, it can be understood that the relative importance of dyadic and systemic factors in predicting adolescent suicidal ideation is similar.

## IMPLICATIONS

As this study underscores the important role of the following features: parent-adolescent communication (particularly mother-adolescent communication), family functioning (particularly mutuality, parental concern, parental control, conflict and harmony) in adolescent suicidal ideation; these features should be considered as the key building blocks in theoretical models, particularly family models, for adolescent suicidal ideation. The present findings are important because there are few theoretical models on the family determinants of adolescent behavior in the Chinese context[35]. As theoretical models on the family determinants of adolescent suicidal ideation are almost nonexistent in the Chinese cultural contexts, the present findings are a pioneering contribution to the literature.

As the present findings suggest that it is important to enhance parent-adolescent communication, several practice possibilities for preventing adolescent suicidal ideation can be considered in this context. First, parallel groups could be considered for both the parents and adolescents enhancing their communication skills[62]. This facilitates reciprocal changes both in the parents and the adolescents. Second, a manual-based parenting program, such as the Systematic Training for Effective Parenting

Program designed by Dinkmeyer and McKay, based on Adlerian psychology, could be introduced to improve the parent-adolescent relationship[63]. A manual-based training program conducted by professionals is suitable for Chinese parents because they tend to listen to authoritative figures and adhere to systematic training. This can facilitate them to learn new concepts and skills, e.g., assertive communication.

However, it should be noted that communication and emotional expressiveness were not emphasized in traditional Chinese families[23]. Parents and adolescents, especially the fathers and the sons, might feel uneasy or embarrassed to express emotional issues. Direct and open expression of feelings, particularly negative feelings, may be interpreted as blaming. Moreover, parents like to use double-bind messages rather than clear and specific statements, and are accustomed to use name-calling and put-down words rather than express their worries and concern.

Based on the above discussion and the literature on Chinese family intervention, the following content for Chinese parent communication groups is suggested. Parents are guided to: (1) respect and trust the adolescents; (2) provide more opportunity as well as a free and relaxing environment for communication; (3) take initiatives to express their concern to the adolescents; (4) be willing to listen to what the adolescents say; look at things from the adolescents' perspectives; (5) reflect on the adolescents' thinking and feelings; (6) guide the adolescents to think out different solutions, evaluate their pros and cons, and let them make decisions; (7) encourage the adolescents to express their thinking and feelings openly, and to raise problems for discussion. The parents should also be guided to avoid ordering, ridiculing, criticizing, provoking, indoctrinating, or nagging the adolescents; dominating the discussion and using double-bind messages. In turn, the Chinese adolescents should also be taught to respect and trust their parents, take initiative to communicate with their parents, express their feelings and thinking, look at things from the parents' perspectives, and try to understand the parents' thinking and feelings.

The results also indicated that family functioning, particularly parental concern, parental control, conflict and harmony, and mutuality, was significantly related to adolescent suicidal ideation. Thus, it is important to enhance parental concern, harmony, and mutuality, while minimizing parental control and conflict within the family. The Positive Parenting Program for parents, which was effective in promoting harmonious family relationships in Chinese families in Hong Kong, could be organized to strengthen family functioning[64]. Nevertheless, care should be taken that excessive parental concern is not mistaken by adolescents for control, so parents should be guided to give an appropriate level of concern to their teenagers. As many mothers get a large part of their life satisfaction from taking care of and controlling their children, they may be quite fearful of losing control over them[65]. Hence, it is important for family practitioners to appreciate the parents' efforts to take care of their children, but also help them to realize the possible negative impacts of excessive control.

From a systemic perspective, family mutuality and cohesion can be enhanced by guiding the family members to love, care, concern, trust, support, and understand one another. They should also be taught to tolerate, forbear, accommodate, and be considerate to one another. In addition, family harmony can be achieved by teaching the family members ways to resolve family conflicts and facilitating couples to enrich their marital relationships. The parents should also be guided to increase parental concern and decrease parental control by understanding the adolescents' perception of concern and control.

## LIMITATIONS

It is noteworthy that there are several limitations of the present study. First, the weaknesses of convenience sampling should be noted. In particular, generalization of the present findings to the secondary school population should proceed with caution. Second, as self-administered questionnaires were used, the information collected was based on the adolescent informants only. If resources permit, the use of multiple informants that know the adolescents would give a clearer picture of the problem area. Third, the present research predominately used quantitative methodology. In addition, a qualitative study can be conducted to better understand the texture of the relationship between the parents and the adolescents in contributing to adolescent suicidal ideation. Fourth, only two family correlates (perceived parent-adolescent communication and family functioning) were selected, based on a review of empirical findings and the theoretical framework. Other personal factors (e.g., self-esteem), familial factors (e.g., parenting style, family rules), school factors (e.g., school performance, relationship with schoolmates), and societal factors (e.g., mass media, social integration) can also be included in future studies of adolescent suicidal ideation. Fifth, the use of a cross-sectional design means that it is not possible to claim a causal relationship between the antecedents and adolescent suicidal ideation. Hence, a longitudinal research design can be used in future studies. Despite these limitations, the present study can be regarded as a stimulating and interesting addition to the literature that has enriched our understanding of the relationship between family processes (perceived parent-adolescent communication and family functioning) and Chinese adolescent suicidal ideation.
